# Reducing Storage-Related Bias in Reproductive Vitamin D Research: Towards a Stable and Reliable Biomarker of Ovarian Vitamin D Status

**DOI:** 10.3390/nu17233783

**Published:** 2025-12-02

**Authors:** Evelin E. Lara-Molina, Jason M. Franasiak, Almudena Devesa-Peiro, Marina López-Nogueroles, Alberto Vázquez, David Amorós, Agustín Ballesteros, Antonio Pellicer, Patricia Sebastian-Leon, Patricia Diaz-Gimeno

**Affiliations:** 1 IVIRMA Global Research Alliance, IVIRMA Barcelona, 08029 Barcelona, Spain; evelin.lara@ivirma.com (E.E.L.-M.);; 2 IVI Foundation, Instituto de Investigación Sanitaria La Fe (IIS La Fe), 46026 Valencia, Spainpatricia.sebastian@ivirma.com (P.S.-L.); 3 IVIRMA Global Research Alliance, Reproductive Medicine Associates of New Jersey, Basking Ridge, NJ 07920, USA; 4 Sidney Kimmel Medical School, Thomas Jefferson University, Philadelphia, PA 19107, USA; 5 Instituto de Investigación Sanitaria La Fe (IIS La Fe), 46026 Valencia, Spain; 6 IVIRMA Global Research Alliance, IVIRMA Roma, 00161 Rome, Italy

**Keywords:** 25-hydroxyvitamin D_3_, 24,25-dihydroxyvitamin D_3_, ovarian vitamin D status biomarkers, assisted reproduction, LC–MS/MS, vitamin D stability, stored biological samples, ovary, follicular fluid, serum

## Abstract

**Background/Objectives:** One of the main reasons for discrepancies in the role of vitamin D in ART could be the measurement of the conventional biomarker 25(OH)D_3_. It is known that this value is affected by multiple factors, such as tissue origin, assay variability, classification criteria, and potential storage-related degradation. In this study, we investigate 24,25(OH)_2_D_3_ as a new biomarker to improve vitamin D assessment in women’s reproductive health, particularly regarding oocyte development. **Methods:** A prospective cohort study including 35 oocyte donors undergoing controlled ovarian stimulation, who were recruited between October and November 2017, was conducted. Vitamin D metabolites were measured at the baseline and after seven months of storage at −80 °C. Paired serum and pooled follicular fluid (FF) samples were collected at oocyte retrieval. 25(OH)D_3_ and 24,25(OH)_2_D_3_ were quantified by ultra-performance liquid chromatography–tandem mass spectrometry (UPLC–MS/MS). Statistical analyses included paired tests (serum vs. FF; baseline vs. stored) and Pearson’s correlations (two-sided α = 0.05). **Results:** At the baseline, the mean serum 25(OH)D_3_ concentration was 91.56 ± 39.01 nmol/L and the mean FF concentration was 58.13 ± 19.55 nmol/L (*p* < 0.0001). Serum 24,25(OH)_2_D_3_ averaged 15.62 ± 10.99 nmol/L, compared with 11.26 ± 6.09 nmol/L in FF (*p* = 0.004). In both fluids, 25(OH)D_3_ and 24,25(OH)_2_D_3_ were strongly correlated (serum R^2^ = 0.92; FF R^2^ = 0.91). Across fluids, the serum–FF correlation was stronger for 24,25(OH)_2_D_3_ (R^2^ = 0.77, *p* <0.0001) than for 25(OH)D_3_ (R^2^ = 0.69, *p* < 0.0001). After seven months of storage, 25(OH)D_3_ concentrations decreased significantly (serum −32%; FF −38%; both *p* < 0.0001), whereas 24,25(OH)_2_D_3_ levels remained stable (serum *p* = 0.24; FF *p* = 0.36). **Conclusions:** Serum 24,25(OH)_2_D_3_ is a more reliable and minimally invasive biomarker for assessing ovarian vitamin D status than the current gold standard, 25(OH)D_3_. Incorporating this metabolite into research studies and storage quality control may improve the reliability of retrospective analyses based on cryopreserved material, contributing to a better understanding of the role of vitamin D in human reproduction.

## 1. Background

Vitamin D is a secosteroid hormone that becomes biologically active as 1,25-dihydroxyvitamin D (1,25(OH)_2_D) after sequential hydroxylations in the liver and kidney. Beyond calcium and phosphate homeostasis, its multiple effects are mediated through the vitamin D receptor, which is expressed in a broad range of tissues, including the ovary, endometrium, placenta, and testis [1,2,3,4]. In the context of female reproductive health, a recent narrative review [5] highlighted that vitamin D status influences multiple processes, including regulation of the menstrual cycle through steroidogenesis [6,7,8], and luteal function, thereby affecting progesterone production [9]. Adequate vitamin D levels have been associated with improved ovarian stimulation response [10] and enhanced fertility outcomes in in vitro fertilization (IVF) [11]. Conversely, vitamin D deficiency has been linked to pregnancy-related complications, such as preeclampsia, gestational diabetes mellitus, and preterm labour [12], as well as reproductive disorders, including infertility, endometriosis, and polycystic ovarian syndrome [13]. However, findings remain inconsistent when 25(OH)D levels are assessed in follicular fluid. Some studies reported no association between vitamin D concentrations in follicular fluid and IVF outcomes such as fertilization rate or embryo quality [14,15,16], whereas others found significant positive correlations between FF 25(OH)D levels and oocyte or embryo quality [17,18]. Vitamin D deficiency is highly prevalent worldwide and has been frequently reported in women undergoing assisted reproductive technology (ART) [19,20,21]. While insufficient sun exposure and poor dietary intake are clear contributors [22], part of this apparent deficiency may be explained by methodological issues in vitamin D measurement. The most abundant circulating metabolite, 25-hydroxyvitamin D (25(OH)D), is the conventional biomarker for assessing vitamin D status [23,24] and to establish supplementation regimens [25]. However, immunoassay-based quantification of 25(OH)D has been shown to present substantial bias—up to 30%—and inter-laboratory variability as high as 15% [26,27,28,29]. Furthermore, discrepancies between biochemical results and clinical phenotypes have been observed: African American populations often present with low serum 25(OH)D yet show low fracture risk [30,31], and individuals with normal parathyroid hormone levels may also present with low 25(OH)D [32]. Such inconsistencies call into question the accuracy of 25(OH)D as a universal marker of vitamin D status.

Other candidate markers, such as free or bioavailable 25(OH)D, have been proposed; however their interpretation is complicated by polymorphisms in the vitamin D-binding protein, which influence ligand binding and assay performance [33]. In addition, pre-analytical factors, including light exposure, freeze–thaw cycles, and storage duration, can alter measured concentrations, yet are not uniformly controlled. Many studies rely on stored serum and follicular fluid, often cryopreserved or lacking detailed information on storage conditions and processing times. Early studies had already warned about stability issues for vitamin D metabolites [34], and more recent data have reinforced concerns regarding storage-related changes in 25(OH)D [35].

Liquid chromatography–tandem mass spectrometry (LC–MS/MS) has therefore emerged as the reference method to address these issues [36,37], with a reported bias of below 5% and coefficients of variation around 10%, consistent with standards from the Vitamin D Standardization Program [38]. Unlike immunoassays, LC–MS/MS avoids interference from protein binding and cross-reactivity with other vitamin D metabolites, thereby offering greater accuracy and comparability [39]. Nevertheless, inconsistencies in defining sufficiency thresholds, namely ≥50 nmol/L according to the Institute of Medicine [40] and >75 nmol/L by the Endocrine Society [41], further complicate interpretation and may explain conflicting reports regarding reproductive outcomes. Moreover, these cut-offs were established primarily to ensure bone health in the general population, without specifically addressing reproductive health or considering potential sex- or age-related differences. Recent updates from the Endocrine Society (2024) further emphasize that vitamin D sufficiency thresholds cannot be universally applied across populations, tissues, or physiological contexts, reinforcing the need for tissue-specific biomarkers, such as 24,25(OH)_2_D_3_, in reproductive research [42].

Among alternative biomarkers, 24,25-dihydroxyvitamin D (24,25(OH)_2_D) has attracted increasing interest. This metabolite, produced through Cytochrome P450 Family 24 Subfamily A Member 1 (CYP24A1)-mediated hydroxylation of 25(OH)D, represents the principal catabolic pathway of vitamin D [43]. Its synthesis in reproductive tissues, such as the human decidua and placenta, was already demonstrated by Weisman et al. (1979) [1], suggesting a potential physiological role in reproductive processes. Both its absolute concentration and the 24,25(OH)_2_D:25(OH)D ratio have been proposed as functional indices of vitamin D status, measurable only by LC–MS/MS. While systemic studies have validated serum 24,25(OH)_2_D as an accurate indicator of vitamin D metabolism [44], its behaviour in the ovary—particularly in follicular fluid—and its stability during long-term storage remain poorly characterised.

## 2. Methods

### 2.1. Study Design and Participants

**Figure 1:**
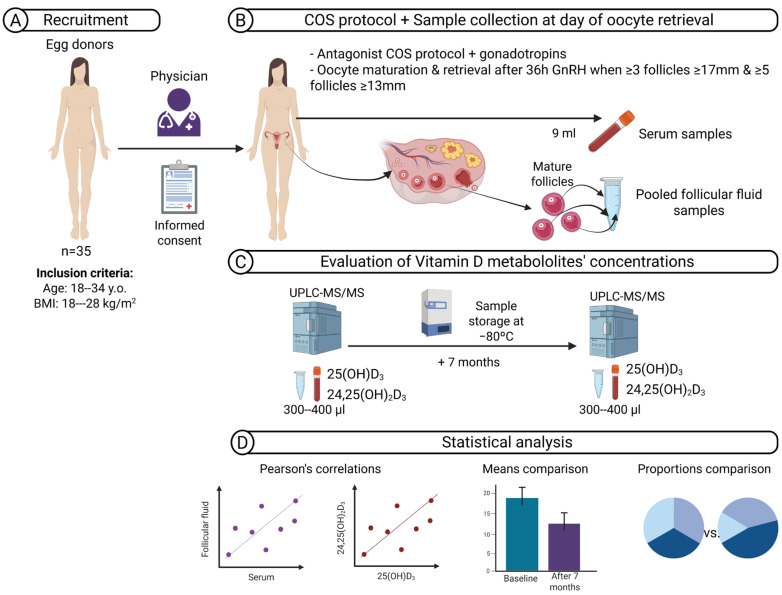
Schematic overview of the study workflow. (**A**) A cohort including 35 healthy women who met the criteria for oocyte donation was recruited. (**B**) Controlled ovarian stimulation (COS) was performed using antagonist protocols until criteria for triggering oocyte maturation were met. On the day of oocyte retrieval, paired serum and pooled follicular fluid (FF) samples were collected. (**C**) Vitamin D metabolites—25-hydroxyvitamin D_3_ (25(OH)D_3_) and 24,25-dihydroxyvitamin D_3_ (24,25(OH)_2_D_3_)—were quantified by ultra-performance liquid chromatography–tandem mass spectrometry (UPLC–MS/MS) at baseline and after seven months of storage at −80 °C. (**D**) Statistical analyses included Pearson’s correlations and means and proportions comparisons. Abbreviations: y.o., years old; BMI, body mass index; COS, controlled ovarian stimulation; GnRH, gonadotrophin-releasing hormone; UPLC–MS/MS, ultra-performance liquid chromatography–tandem mass spectrometry.

The study is schematised in Figure 1 and was designed to investigate whether serum 24,25(OH)_2_D_3_ provides a more reliable reflection of ovarian vitamin D status than 25(OH)D_3_, using paired serum and follicular fluid samples. Because reproductive research frequently relies on cryopreserved and stored material, we also examined the stability of both metabolites after prolonged storage at −80 °C to assess their reliability as biomarkers in studies based on stored samples.

This was a prospective cohort study including 35 healthy women who met the criteria for oocyte donation. Recruitment took place in autumn, between October and November 2017. Inclusion criteria were ages 18–34 years and a body mass index (BMI) of 18–28 kg/m^2^ (Figure 1A). All women underwent controlled ovarian stimulation using a Gonadotropin-Releasing Hormone (GnRH) antagonist protocol with recombinant or urinary follicle-stimulating hormone (FSH), human menopausal gonadotrophin, or a combination, at doses determined by the treating physician. Final oocyte maturation was triggered with a GnRH agonist when ≥3 follicles of ≥17 mm and ≥5 follicles of ≥13 mm were observed. On the day of oocyte retrieval, 9 mL of peripheral blood was collected before follicular aspiration. Samples were immediately placed in light-protected tubes to prevent degradation, as indicated by previous reports [34]. After centrifugation (1500–2000× *g*, 10 min), serum was aliquoted. Follicular fluid from mature follicles was pooled per donor, centrifuged to remove cells, and aliquoted under the same conditions. (Figure 1B). All serum and follicular fluid (FF) samples were frozen at −80 °C until analysis. Baseline analyses were performed within one month after sample collection. A second measurement was performed after seven months of storage at −80 °C (Figure 1C). Finally, different statistical analyses were performed to assess correlations between metabolite values in serum and FF and between them. In addition, mean concentrations and different vitamin D classifications were compared (Figure 1D).

### 2.2. Measurement of 25-Hydroxyvitamin D_3_ Concentration and Classification of Vitamin D Status

Serum and FF concentrations of 25(OH)D_3_ and 24,25(OH)_2_D_3_ were measured by ultra-performance liquid chromatography–tandem mass spectrometry (UPLC–MS/MS), as previously described [36,37]. Internal standards (25-hydroxyvitamin D_3_-d_6_ and (24R),24,25-dihydroxyvitamin D_3_-d_6_; Santa Cruz Biotechnology, Dallas, TX, USA) were used. The same samples were re-analysed after seven months of storage at −80 °C (Figure 1C).

Serum 25(OH)D_3_ concentrations were classified according to two clinical guidelines: the Institute of Medicine (IOM) thresholds [40], defining sufficiency as ≥50 nmol/L, insufficiency as 30–50 nmol/L, and deficiency as <30 nmol/L, and the Endocrine Society criteria [41], defining sufficiency as ≥75 nmol/L, insufficiency as 50–75 nmol/L, and deficiency as <50 nmol/L.

These classifications were used to compare the distribution of vitamin D status in the cohort and to evaluate potential discrepancies between both systems after sample storage.

### 2.3. Statistical Analysis

Paired *t*-tests were applied to compare serum vs. FF concentrations and baseline vs. stored values. Pearson’s correlations were calculated for metabolite associations. Kappa’s agreement coefficient was estimated using the vcd R package (version 1.4.8) [45] to assess concordance between the two classification systems. Agreement values were interpreted as follows: none (0–0.20), minimal (0.21–0.39), weak (0.40–0.59), moderate (0.60–0.79), strong (0.80–0.90), and almost perfect (>0.90) [46]. Fisher’s exact test was applied to evaluate the difference in classifications for serum 25(OH)D_3_ between the baseline and after seven months at −80 °C. All statistical analyses were performed using paired comparisons between serum and follicular fluid samples obtained from the same participants, along with before and after long-term storage, ensuring the removal of inter-individual variability and enhancing the power to detect intra-individual differences. Statistical significance was set at *p* ≤ 0.05. All analyses were performed using R version 4.4.3 [47] (Figure 1D).

## 3. Results

### 3.1. Characteristics of the Study Population

Of the 35 oocyte donors initially recruited, one was excluded due to technical failure in serum vitamin D measurement, leaving 34 women for analysis. The cohort presented a mean age of 25.4 ± 4.6 years and a mean BMI of 22.9 ± 2.7 kg/m^2^. The ovarian response to stimulation has an average of 28.2 ± 11.1 follicles aspirated and 23.3 ± 9.1 mature oocytes retrieved. Additional cycle characteristics, including menstrual cycle length, anti-Müllerian hormone (AMH) levels, and stimulation duration and hormone values on the day of triggering, are shown in Table 1. These results confirm that the study population represented a group of healthy young women with normal ovarian function and without clinical confounders.

### 3.2. Serum Vitamin D Metabolites as Biomarkers of Follicular Fluid Concentrations

**Figure 2:**
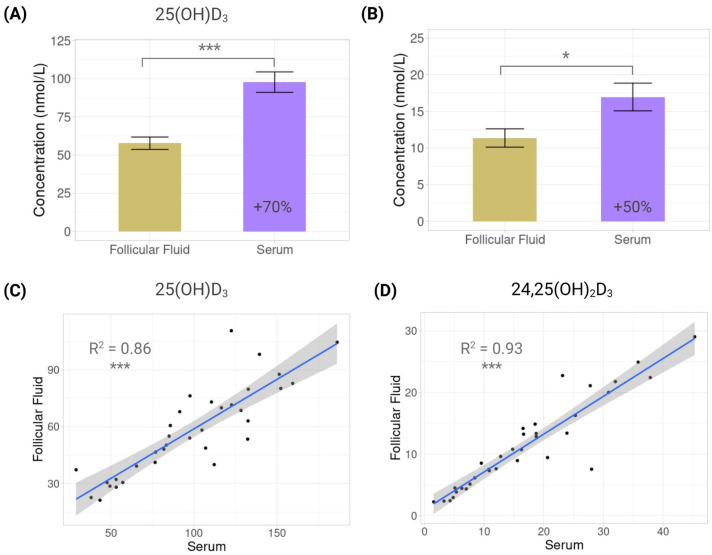
Vitamin D metabolites in serum and follicular fluid. Bar plots showing mean concentration differences of vitamin D metabolites in serum and follicular fluid: (**A**) 25-hydroxyvitamin D_3_ (25(OH)D_3_); (**B**) 24,25-dihydroxyvitamin D_3_ (24,25(OH)_2_D_3_). Scatterplots showing correlations between serum and follicular fluid: (**C**) 25-hydroxyvitamin D_3_ (25(OH)D_3_); (**D**) 24,25-dihydroxyvitamin D_3_ (24,25(OH)_2_D_3_). Each dot represents one individual (*n* = 34). Regression line with 95% confidence interval is displayed. *: *p* < 0.05; ***: *p* < 0.001.

At the baseline, serum concentrations of 25(OH)D_3_ were significantly higher than those in follicular fluid (97.7 ± 39.0 vs. 57.7 ± 23.7 nmol/L, *p* < 0.0001), with serum levels 69.3% higher than FF levels (Figure 2A). A similar pattern was observed for 24,25(OH)_2_D_3_, with serum levels at 16.96 ± 11.0 nmol/L and FF at 11.3 ± 7.3 nmol/L (*p* = 0.0165) and with serum levels that were 49.1% higher than FF levels (Figure 2B). Finally, concentrations of 25(OH)D_3_ were 5.7 and 5.1 times higher than 24,25(OH)_2_D_3_ in serum and follicular fluid, respectively.

Despite these differences, cross-fluid correlations showed that serum values were predictive of FF concentrations, but the relationship was stronger for 24,25(OH)_2_D_3_ (R^2^ = 0.93, *p* < 0.0001) than for 25(OH)D_3_ (R^2^ = 0.86, *p* < 0.0001) (Figure 2C,D).

**Figure 3:**
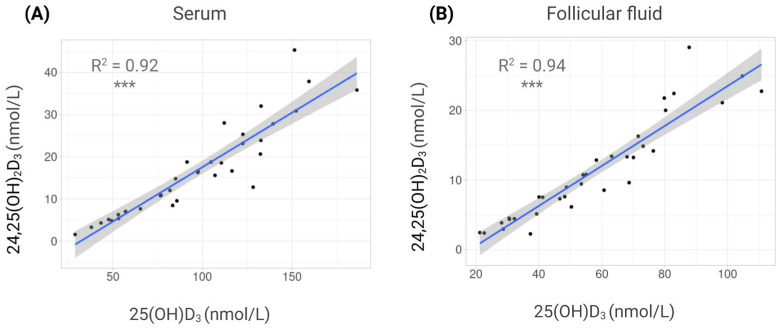
Correlations between vitamin D metabolites in different fluids. Scatterplots showing correlations between 25-hydroxyvitamin D_3_ (25(OH)D_3_) and 24,25-dihydroxyvitamin D_3_ (24,25(OH)_2_D_3_) concentrations. (**A**) Serum. (**B**) Follicular fluid (FF). Each dot represents one individual. Regression line with 95% confidence interval is displayed. ***: *p* < 0.001.

Finally, both metabolites were strongly correlated in each fluid, showing the same tendency: in serum, R^2^ = 0.92 (*p* < 0.0001), and in FF, R^2^ = 0.94 (*p* < 0.0001) (Figure 3).

### 3.3. Effect of Long-Term Storage on Vitamin D Metabolite Levels

**Figure 4:**
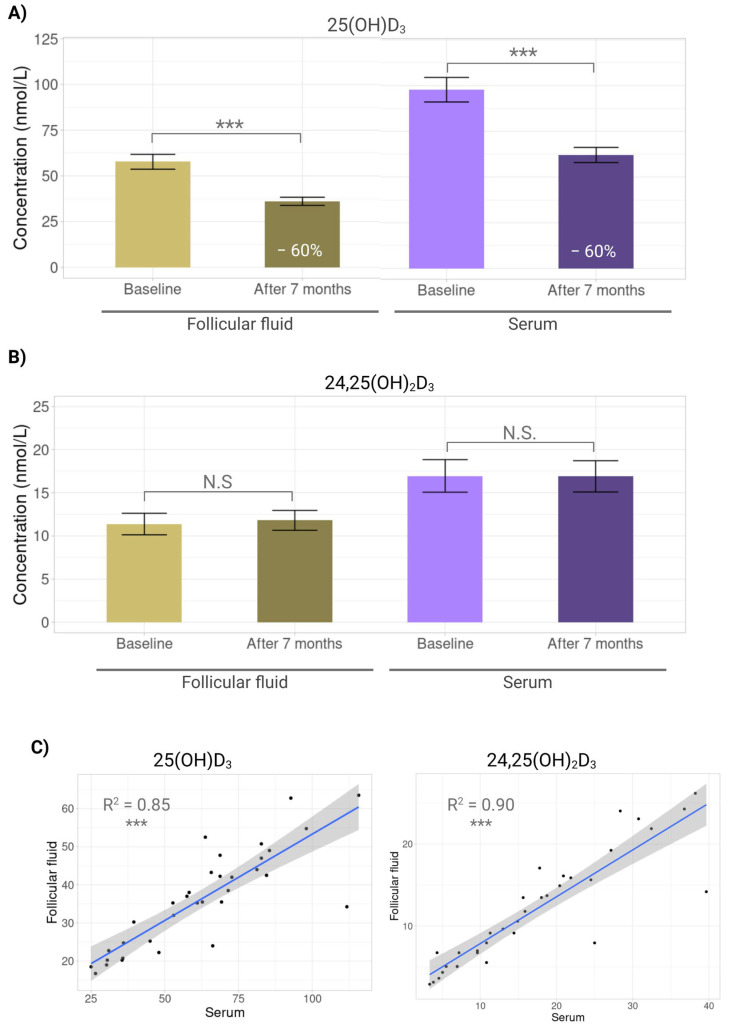
Effect of long-term storage on vitamin D metabolites. Bar plots showing mean concentration differences of vitamin D metabolites in serum and follicular fluid at baseline and after seven months storage at −80 °C. (**A**) 25-hydroxyvitamin D_3_ (25(OH)D_3_). (**B**) 24,25-dihydroxyvitamin D_3_ (24,25(OH)_2_D_3_). (**C**) Scatterplots showing correlations between serum and follicular fluid for 25-hydroxyvitamin D_3_ (25(OH)D_3_) and 24,25-dihydroxyvitamin D_3_ (24,25(OH)_2_D_3_) after 7 months storage at −80 °C. N.S: No significant (*p* > 0.05); ***: *p* < 0.001.

After seven months at −80 °C, concentrations of 25(OH)D_3_ declined markedly in both fluids. In FF, levels fell significantly from 57.7 ± 23.7 to 36.1 ± 12.9 nmol/L (*p* < 0.0001), which represents a decrease of 59.8%, and in serum, from 97.7 ± 39.0 to 62.1 ± 24.1 nmol/L (*p* < 0.0001), which represents a decrement of 57.5% (Figure 4A). By contrast, 24,25(OH)_2_D_3_ remained stable, with no significant changes in FF (11.4 ± 7.3 vs. 11.8 ± 6.8 nmol/L, *p* = 0.8043) or serum (17.0 ± 11.0 vs. 16.9 ± 10.6 nmol/L, *p* = 0.9825) (Figure 4B). These findings confirm that 25(OH)D_3_ is storage-sensitive, whereas 24,25(OH)_2_D_3_ is robustly stable. Despite the decline in 25(OH)D_3_, post-storage cross-fluid correlations remained high for both metabolites (25(OH)D_3_ R^2^ = 0.85, *p* < 0.0001; 24,25(OH)_2_D_3_ R^2^ = 0.90, *p* < 0.0001) (Figure 4C).

### 3.4. Inconsistency in Conventional Vitamin D Status Classification

When the baseline serum 25(OH)D_3_ was classified using the Endocrine Society criteria, most women (73.53%) were sufficient, 11.76% insufficient, and 14.71% deficient. In contrast, according to the Institute of Medicine (IOM) thresholds, most women (85.29%) were sufficient, 11.76% insufficient, and 2.94% deficient. These two classifications showed minimal agreement (Kappa value = 0.3369), illustrating how the choice of classification system substantially alters the apparent prevalence of sufficiency when applied to the same cohort.

**Figure 5:**
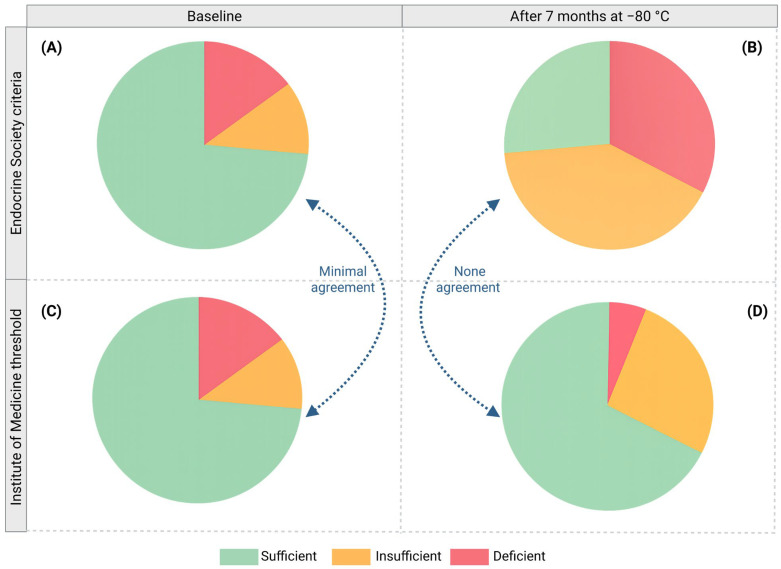
Inconsistency of conventional vitamin D status classification. Classification of serum 25(OH)D_3_ levels according to two different systems: Endocrine Society criteria (**A**,**B**) and Institute of Medicine (IOM) thresholds (**C**,**D**) at baseline (**A**,**C**) and after seven months of storage at −80 °C (**B**,**D**). Both classifications were compared according to their Kappa value.

These differences were more noticeable when serum 25(OH)D_3_ was evaluated after seven months at −80 °C. According to the Endocrine Society criteria, 26.47% of women were sufficient, 41.18% insufficient, and 32.35% deficient, while according to the IOM thresholds, most women remained sufficient (67.65%), 26.47% insufficient, and 5.88% deficient. Therefore, after seven months at −80 °C, both classifications showed no agreement (Kappa value = 0.0237) (Figure 5).

Furthermore, the proportions of both serum 25(OH)D_3_ classifications were significantly different between the baseline and after seven months at −80 °C (IOM thresholds: *p* = 0.0005; Endocrine Society criteria: *p* < 0.0001).

## 4. Discussion

This work highlights the problem of vitamin D measurement in women’s reproductive health and, in particular, provides evidence of a more reliable vitamin D biomarker for ovarian research. In this study, we found that the serum concentration of the proposed vitamin D metabolite, 24,25(OH)_2_D_3_, correlated more strongly with follicular fluid (FF) concentrations than did 25(OH)D_3_, the current gold standard, and that 24,25(OH)_2_D_3_ remained stable after long-term storage at −80 °C, whereas 25(OH)D_3_ decreased significantly. Taken together, these findings suggest that 24,25(OH)_2_D_3_, the proposed vitamin D metabolite, may serve as a more reliable indicator of ovarian vitamin D status than 25(OH)D_3_, especially when analyses are performed on stored samples.

The association between vitamin D and reproductive outcomes has been the focus of considerable debate. Some studies and meta-analyses have reported higher clinical pregnancy or live birth rates among women with sufficient vitamin D levels [21,48,49,50,51], whereas findings from other studies did not support a significant association [49,52,53,54,55,56]. Such conflicting results have fuelled ongoing controversy regarding the clinical relevance of vitamin D in ART. Several explanations have been proposed for these discrepancies, including differences in study populations, variable adjustment for confounders, and—importantly—heterogeneity in how vitamin D status is measured. Immunoassay-based quantification of 25(OH)D_3_ is prone to matrix effects, cross-reactivity, and inter-laboratory variability [37,57]. In addition, cut-offs applied to define sufficiency differ between guidelines: the Institute of Medicine [40] proposes ≥50 nmol/L, while the Endocrine Society recommends >75 nmol/L [41]. As shown in our data, prevalence estimates of deficiency and sufficiency change markedly depending on which threshold is used, and this lack of agreement is more noticeable after long-term storage at −80 °C. This variability illustrates the limitations of relying solely on serum 25(OH)D_3_ for classification in both clinical and research contexts. Standardisation initiatives have attempted to address these classification issues. Large population-based surveys applying liquid chromatography–tandem mass spectrometry (LC–MS/MS) and aligned with the Vitamin D Standardisation Program have consistently reported higher mean serum 25(OH)D levels and lower prevalence of deficiency than studies relying on immunoassays [22,58,59,60]. These findings suggest that vitamin D deficiency may have been systematically overestimated. Our results add another layer to this problem by showing that 25(OH)D_3_ is also highly sensitive to cryopreservation and long-term storage, declining by approximately one third in both serum and follicular fluid after seven months at −80 °C. This raises the possibility that at least part of the variability in the literature, much of which is based on the use of stored samples, may be due to differences in pre-analytical handling and storage conditions.

By contrast, 24,25(OH)_2_D_3_ demonstrated remarkable stability, with minimal changes observed after the same storage interval. Its stronger serum–FF correlation further indicates that it may more faithfully represent ovarian vitamin D status and can be measured through a minimally invasive approach. Biologically, this is plausible: 24,25(OH)_2_D_3_ is the principal catabolic product of 25(OH)D_3_ via CYP24A1 and is therefore tightly linked to systemic vitamin D metabolism [39,43,61]. Its stability may reflect differences in molecular susceptibility to degradation during storage, while its tighter coupling with follicular fluid could relate to its role as a functional readout of enzymatic activity rather than absolute substrate concentration. Genetic variation in vitamin D binding protein can further influence metabolite distribution, and metabolites less dependent on binding dynamics may provide more reproducible measures across individuals. It is important to note that the present study was not intended to validate a new analytical method, as both 25(OH)D_3_ and 24,25(OH)_2_D_3_ were measured using an already established LC–MS/MS platform. Instead, our work aimed to identify and propose 24,25(OH)_2_D_3_ as a more stable and reliable biomarker of vitamin D status for reproductive research. Future population-based studies will be required to establish reference ranges and assess its predictive value for reproductive outcomes.

The importance of storage effects on vitamin D metabolites was recently highlighted by Ko et al., 2025 [35], who demonstrated that serum 25(OH)D concentrations decrease significantly after storage at −80 °C. Their study raised the concern that a misclassification of vitamin D status could occur in research based on archived samples. Our data are consistent with these findings and extend them by showing that declines also occur in follicular fluid—an ovarian compartment directly relevant to oocyte development—and by demonstrating that 24,25(OH)_2_D_3_ remains unaffected by this storage effect. Furthermore, we show that serum 24,25(OH)_2_D_3_ provides a stronger reflection of FF levels than serum 25(OH)D_3_, which is an important methodological consideration for studies using serum as a surrogate for ovarian vitamin D status.

Several mechanisms could underlie the differential behaviour observed between these metabolites. The decline in 25(OH)D_3_ may reflect its greater chemical instability during freezing and thawing, while the stability of 24,25(OH)_2_D_3_ could relate to its different molecular structure or binding properties. The stronger serum–FF correlation observed for 24,25(OH)_2_D_3_ may also be due to its position in the metabolic pathway, functioning as a product of regulated catabolism rather than as a substrate whose concentration is influenced by diet, supplementation, and storage artefacts.

The clinical and wider implications of these findings merit discussion. For investigators relying on retrospective analyses of biobanked samples, the use of 24,25(OH)_2_D_3_, preferably measured by LC–MS/MS, may substantially improve the reliability and accuracy of results. Our results also caution against overinterpretation of 25(OH)D_3_ data from stored specimens, which may underestimate true values. From a clinical perspective, however, 25(OH)D_3_ will remain the pragmatic biomarker of choice because it is widely available, inexpensive, and recommended in guidelines [41]. The adoption of 24,25(OH)_2_D_3_ into routine clinical care is limited by the cost and technical requirements of LC–MS/MS, and the use of this metabolite would necessitate the development of new classification thresholds, since its levels in both serum and FF differ from those of 25(OH)D_3_. Our findings should therefore be interpreted primarily in the research context—as a methodological refinement to enhance our understanding of vitamin D physiology—rather than as a call to change clinical practice. In this context, our study offers a controlled experimental setting to examine vitamin D metabolism and to identify metabolites that may provide more stable and physiologically relevant information about ovarian vitamin D status. These results provide evidence supporting 24,25(OH)_2_D_3_ as a good candidate for studying the ovarian vitamin D profile, as we propose a more reliable metabolite between serum and follicular fluid as well as after months of storage, which is common in molecular research. However, for broader implications in clinical practice, it should be further tested in patient populations and associated with reproductive outcomes.

Although exploratory in nature, our study has some strengths worth noting. We assessed two metabolites in both serum and follicular fluid, used LC–MS/MS as a reference method, and explicitly tested the effect of long-term storage, which is often overlooked. The homogeneous cohort of young, healthy donors reduced confounding by age, BMI, and comorbidities, while collection only during the autumn season minimised seasonal variation in sun exposure that could otherwise influence vitamin D levels [62]. Together, this provided a clean model to explore methodological questions.

Limitations include the restriction to oocyte donors, which limits generalisability to infertile populations, older women, or those with comorbid conditions. Moreover, our design did not include a direct assessment of reproductive outcomes, which prevents drawing conclusions about clinical efficacy. Future research should explore whether 24,25(OH)_2_D_3_, alone or in combination with 25(OH)D_3_, predicts outcomes of clinical importance, such as oocyte competence, embryo quality, implantation, and live birth. Validation in larger and more diverse populations is needed, including infertile women; those with polycystic ovary syndrome, diminished ovarian reserve, and obesity; and across different ancestries. Prospective trials should incorporate standardised protocols for sample handling, storage, and analysis and should explore whether incorporating 24,25(OH)_2_D_3_ into multiparametric biomarker panels alongside other ovarian markers improves predictive models in ART.

In summary, this study contributes to clarifying a methodological source of variability in vitamin D research, which is essential for understanding the role of vitamin D in female fertility. By showing that serum 24,25(OH)_2_D_3_ remains stable during long-term storage and correlates more closely with follicular fluid than 25(OH)D_3_, we provide evidence that this metabolite may offer added value as a research biomarker, particularly for retrospective studies based on archived samples. Although its measurement by high-performance liquid chromatography–tandem mass spectrometry (LC–MS/MS) remains challenging in clinical settings, the stability and stronger ovarian relevance of 24,25(OH)_2_D_3_ suggest that its incorporation into reproductive research may help overcome storage-related limitations and improve the reliability of studies based on archived samples.

## Figures and Tables

**Table 1:** Baseline characteristics of the study population.

Variable	Mean ± SD
Age (y.o.)	25.43 ± 4.57
Body mass index (kg/m^2^)	22.88 ± 2.67
Menstrual cycle length (d)	29.74 ± 6.19
Antral follicular count	23.29 ± 5.11
Anti-Müllerian hormone (ng/mL)	1.90 ± 0.92
E_2_ on triggering day (pg/mL)	2956.5 ± 1515.6
Progesterone on triggering day (ng/mL)	1.14 ± 0.57
Controlled ovarian stimulation duration (d)	10.57 ± 1.07
FSH total dosage (IU)	1509.29 ± 784.55
HMG total dosage (IU)	140.70 ± 540.77
Total follicles aspirated	28.17 ± 11.07
Total matured (metaphase II) oocytes retrieved	23.29 ± 9.13

Clinical and demographic features of oocyte donors were included in the study (*n* = 35). Values are presented as the mean ± standard deviation (SD) unless indicated otherwise. Abbreviations: SD, standard deviation; y.o., years; BMI, body mass index; E_2_, oestradiol; FSH, follicle-stimulating hormone; HMG, human menopausal gonadotrophin.

## Data Availability

The clinical datasets generated and analysed during the current study have not been deposited in a public repository due to patient privacy and ethical restrictions but are available from the corresponding author on reasonable request (P.D-G and P. S-L.). Metabolite concentrations are included in Supplementary File S1.

## Supplementary Materials

The following supporting information can be downloaded at: https://www.mdpi.com/article/10.3390/nu17233783/s1, Supplementary File S1 (Datos.xlsx): Metabolite concentrations. Concentrations of vitamin D metabolites—25-hydroxyvitamin D_3_ (25(OH)D_3_) and 24,25-dihydroxyvitamin D_3_ (24,25(OH)_2_D_3_)—quantified by ultra-performance liquid chromatography–tandem mass spectrometry (UPLC–MS/MS) at baseline and after seven months of storage at −80 °C for serum and follicular fluid.
